# An improved HPAEC-PAD method for the determination of D-glucuronic acid and 4-O-methyl-D-glucuronic acid from polymeric and oligomeric xylan

**DOI:** 10.1186/s12896-024-00931-9

**Published:** 2024-12-12

**Authors:** Savvina Leontakianakou, Carl Grey, Eva Nordberg Karlsson, Roya R. R. Sardari

**Affiliations:** Division of Biotechnology, Department of Chemistry, Lund University, PO Box 124, 22100 Lund, Sweden

**Keywords:** GlcA, MeGlcA, Hardwood, Polysaccharides, Oligosaccharides

## Abstract

Glucuronic acid (GlcA) is an abundant substituent in hardwood xylan, and it is often found in its methylated form as methyl glucuronic acid (MeGlcA). GlcA and MeGlcA are sugar acids, bound to the xylose backbone at position O-2, and their presence can affect the digestibility of the polymer. Currently, detection of released GlcA or MeGlcA from synthetic substrates such as pNP-glucuronic acid can be achieved with colorimetric assays, whereas analysis from natural substrates such as xylan is more complicated. High performance anion exchange chromatography with pulsed amperometric detection (HPAEC-PAD) with an isocratic elution profile has been proposed for quantification of uronic acids in acid-hydrolysed wood samples. However, achieving sufficient separation for comprehensive analysis of hardwood-related xylan components, particularly MeGlcA remains challenging with this methodology. This study offers modified protocols for improved separation by introducing gradient elution profiles to effectively separate hydrolysed hardwood-related compounds, including MeGlcA, and GlcA within a single analytical run. The method showed excellent reproducibility and a standard curve of MeGlcA assured first order linearity in a wide range of concentrations, making the method excellent for quantification.

**Supplementary Information:**

The online version contains supplementary material available at 10.1186/s12896-024-00931-9.

## Highlights

•GlcA and MeGlcA affect the enzymatic digestibility of hardwood xylan.

•HPAEC-PAD methodology developed for identification and quantification of MeGlcA release from xylan substrates.

•MeGlcA from xylan hydrolysate fractionated using HPAEC-PAD and verified by ESI-MS.

•Separation limitations by HPAEC-PAD addressed by shifting from isocratic to gradient conditions.

•Sodium acetate gradient regulated for quantification of MeGlcA from oligomeric substrates.

**Graphical Abstract:**

**Supplementary Information:**

The online version contains supplementary material available at 10.1186/s12896-024-00931-9.

## Graphical abstract

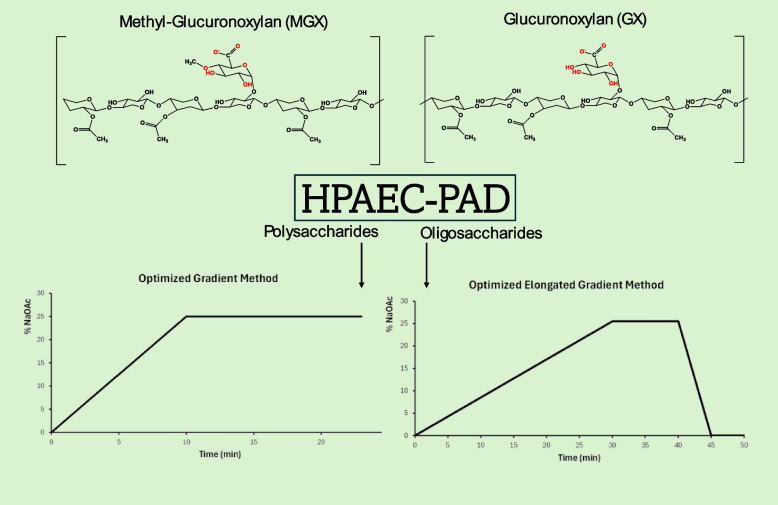

## Introduction

Hemicellulose is a major component of plant cell walls, alongside cellulose, pectin, glycoproteins, and lignin [1, 2]. Xylan is the most abundant type of hemicellulosic polymer. The backbone consists of xylosyl units connected by β-(1,4) glycosidic bonds, with various substitutions. Depending on the origin of xylan, the main substituents include glucuronic acid (GlcA), 4-O-methyl-glucuronic acid (MeGlcA) or both [(Me)GlcA] attached to the O-2 position of the backbone residues [3, 4], and α-L-arabinose at positions O-2 and/or O-3 [5]. The arabinose unit can potentially be linked to ferulic acid via an ester bond at the O-5 position. Additionally, xylan can be heavily acetylated, at positions O-2 and/or O-3 [3, 6]. These diverse substitutions can impact the folding of xylan and its interactions with other cell wall components. They also significantly affect the properties and functionality of xylan [7, 8]. Their presence is notable in various types of xylan including those derived from hardwood, softwood, agricultural residues, and grasses. These substitutions modulate xylan characteristics such as solubility and rheological properties, as well as influence enzymatic digestibility [9, 10]. Therefore, the detection and quantification of these substituents are of importance for evaluating the properties of the polymer, and exploring its diverse potential applications [11, 12].

To study the substituents, xylan often needs to be hydrolysed. This can be achieved by non-specific acid hydrolysis, or by enzymatic hydrolysis that attack specific structures in the xylan. (Me)GlcA is a significant substituent of xylan in hardwood, with its degree of substitution ranging from 1 in 10 to 1 in 3 [13]. Specific characteristics can thus be achieved for this type of xylan by modifying the degree of (Me)GlcA-substitution via use of specific enzymes (GH115 glucuronidases) that selectively release (Me)GlcA from xylan without breaking down the entire polymer [14].

When screening for novel enzyme candidates, *p*NP-glucuronic acid and similar synthetic substrates are valuable tools, providing a convenient way to identify carbohydrate-active enzymes that hydrolyse (Me)GlcA substituents [15–18]. However, they do not represent the complexity of natural xylan substrates, where carbohydrate-active enzymes often interact with adjacent sugar residues within the substrate backbone. Such interactions are absent in synthetic substrates like *p*NP-glucuronic acid, as the only backbone is the para-nitrophenyl group [19], which is short and may not be recognized by the binding site of certain carbohydrate-active enzymes, resulting in misleading activity data.

To increase relevance, two alternative methods have been proposed: spectrophotometric determination after coupled enzyme reactions, and detection of uronic acids by High Performance Anion Exchange Chromatography with a Pulsed Amperometric Detection (HPAEC-PAD).

In the coupled enzyme reaction method, release of (Me)GlcA from a natural substrate is combined with an oxidoreductase reaction, in which the reduction of NAD^+^ to NADH is stoichiometrically equivalent to the available released (Me)GlcA and can be monitored spectrophotometrically [18, 19]. Briefly, the glucuronidase (the primary enzyme), hydrolyses (Me)GlcA from the natural substrate. (Me)GlcA is then oxidised by the secondary enzyme (a dehydrogenase), together with the added cofactor NAD ^+^ , forming NADH as the final detectable product. However, detection of a secondary product (NADH) is an indirect method that may in some circumstances lead to underestimation of the primary enzyme's activity.

The other alternative method is HPAEC-PAD, which is extensively used for direct detection of uronic acids, monosaccharides, and oligosaccharides, offering distinct advantages over spectrophotometric methods in carbohydrate analysis [20, 21]. HPAEC operates under alkaline conditions, alongside a PAD detector. This setup, at high pH (> 12), allows neutral carbohydrates to undergo partial ionization of their hydroxyl groups, thereby allowing the chromatographic separation and detection capabilities for underivatized oligo- and polysaccharides, up to specific degrees of polymerization [22, 23]. Basumallick and Rohrer [24] presented a HPAEC-PAD method (here termed “conventional method”) utilizing an isocratic elution profile for determination and quantification of uronic acids in acid-hydrolysed wood samples. However, achieving comprehensive analysis of the mixture of hardwood-related compounds consisting of xylose, arabinose, GlcA, and specifically MeGlcA along with incompletely hydrolysed oligosaccharides in a single analytical run remains challenging.

In this study, we address this problem by presenting a modified analytical HPAEC-PAD method utilizing gradient elution to achieve efficient separation of arabinose (A), xylose (X), xylobiose (X2), xylotriose (X3), xylotetraose (X4), xylopentaose (X5), xylohexaose (X6), MeGlcA, and GlcA in a single analytical run.

## Materials and Methods

### Materials

Most of the chemicals and reagents were purchased from Merck (Sigma -Aldrich), Sweden, unless otherwise stated. (Me)GlcA was obtained from Biosynth. Standards of arabinose and xylose as monosaccharides, along with xylobiose (X2), xylotriose (X3), xylotetraose (X4), xylopentaose (X5), xylohexaose (X6) as xylan oligosaccharides, were obtained from Megazymes. Also, beechwood xylan and 4-O-Methyl-α-D-Glucuronyl- xylotetraose (XUXX) were purchased from Megazyme. Ultrapure water (Milli-Q grade) was used for all experiments mentioned in this study.

### Methods

#### Enzymatic Hydrolysis of Beechwood Xylan and XUXX

Beechwood xylan and XUXX were suspended separately to a final concentration of 5 g/L in citrate–phosphate buffer pH 6. The suspensions were incubated with 0.01 g/L GH115 α-Glucuronidase at 38 °C for 24 h. Samples were collected in frequent intervals to monitor the hydrolysis progress. After the incubation, the samples were boiled for 10 min to terminate the reaction, followed by proper dilution in ultrapure water and filtering through 0.22 μm polypropylene filters for further analysis.

#### HPAEC-PAD chromatography using Dionex (ISC 6000) system

All analysis in this study were performed by HPAEC-PAD (Thermo Fisher Scientific, Waltham, USA) using Dionex (ISC 6000) system. A CarboPac PA200 analytical column (250 mm × 3 mm, 5.5 µm particle size) equipped with a guard column (50 mm × 3 mm) was used for the analysis. The separation was achieved with three different eluents known as A, B, and C containing ultrapure water, NaOH + NaOAc, and NaOH, respectively. Depending on the analysis method, different concentrations of NaOH and NaOAc with different gradients were used. The temperatures of the column and compartment were maintained at 30 °C throughout the run time. The analytes were detected with an ED40 electro-chemical detector (Thermo Fisher Scientific) and the reference electrode was in PdH mode.

##### Conventional analysis method with isocratic elution

Conventional analysis of the reference compounds (from Sect. "Materials") and the reaction mixture in the samples before and after enzymatic hydrolysis, was performed using two eluents: B (100 mM NaOH containing 1 M NaOAc) and C (100 mM NaOH). Separation occurred during 30 min running time using isocratic condition at the flow rate of 0.5 mL/min. The eluent composition B was maintained at 2% in the first 18 min, then increased to 50% in 1 min and maintained at 50% for 4 min to wash the column with a higher concentration of acetate (500 mM) before equilibrating to initial conditions. The eluent composition then, decreased to 2% in 1 min which was maintained until the end of the run (Table 1).

**Table 1:** Comparison of elution profiles and conditions for conventional, optimized gradient, and elongated gradient methods in HPAEC-PAD Analysis. The mobile phase composition for the isocratic conventional method was B: 100 mM NaOH containing 1 M NaOAc and, C: 100 mM NaOH, and for the gradient methods: A ultrapure water, B: 200 mM NaOH containing 400 mM NaOAc and, C: 200 mM NaOH

Conventional Method			Optimized Gradient Method				Elongated Gradient Method			
Time (min)	B (%)	C (%)	Time (min)	A (%)	B (%)	C (%)	Time (min)	A (%)	B (%)	C(%)
0.0	2	98	-1.0	50	0	50	-1.0	50	0	50
18.0	2	98	0.0	50	0	50	0.0	50	0	50
18.1	50	50	10	50	25	25	30	50	25	25
22.0	50	50	10.1	50	25	25	40	50	25	25
22.1	2	98	23.0	50	25	25	45	50	0	50
30.0	2	98					50	50	0	50

##### Optimized gradient analysis method with gradient elution

In this method, the reference compounds and the reaction mixture in the samples before and after enzymatic hydrolysis were analyzed using three eluents; A (ultrapure water), B (200 mM NaOH containing 400 mM NaOAc) and C (200 mM NaOH). Eluent A was set at 50% throughout the run time. Separation occurred during 23 min running time using gradient conditions at the flow rate of 0.5 mL/min. The gradient (B-C) was increased from 0 to 25% in the first 10 min and was maintained 25% until the end of the run (Table 1).

##### Elongated analysis method with slow gradient

The set up was similar to the optimized gradient analysis method described in Sect. (Optimized gradient analysis method with gradient elution) with the only difference being that the linear gradient (B-C) from 0 to 25% was achieved at a much slower pace, reached in 30 min, and then kept constant for 10 min to wash the column. After that, the gradient (B-C) was decreased to 0% in 5 min and maintained at 0% for 5 min until the end of the run to re-equilibrate the column for the next injection. Therefore, the total run time was increased from 23 to 50 min (Table 1).

#### Internal fraction collection in HPAEC-PAD for product isolation

Internal fraction collection was set up to the HPAEC-PAD system to isolate and identify the product of interest. A Dionex ERD 500 4 mm, electrolytically regenerated desalter (ThermoFisher Scientific) was coupled at the detector’s outlet assuring the removal of Na^+^ ions in exchange for H^+^, to ensure that all samples were suitable for MS analysis. During desalting, ultrapure water, with a flow rate of 4.2 mL/min was used as regenerant with electrolysis current set at 250 mA.

#### Electrospray Ionization Mass Spectrometry Analysis (ESI–MS)

The desalted HPAEC-PAD fraction underwent MS analysis in negative mode using electrospray ionization (ESI). Prior to injection, samples were diluted to a 1:1 ratio with aqueous solution of 80% LC-Mass grade methanol. Subsequently, samples were directly injected into the linear-ion-trap mass spectrometer LTQ Velos-Pro (ThermoFisher Scientific) at a flow rate of 10 μl/min. The ESI source operated at 150 °C with sheath gas flow and auxiliary gas flow set at 7 and 5 arbitrary units, respectively. The spray voltage was established at -3.00 kV, and the capillary temperature maintained at 250 °C. The MS^n^ spectra were collected in negative mode, and the experiments were conducted using collision-induced dissociation (CID) at a collision energy of 35.00 eV. For background spectra observation, ultrapure water was utilized, and 10μM glucuronic acid served as standard.

## Results & Discussion

### Enzymatic Hydrolysis of Beechwood Xylan and XUXX

**Fig. 1:**
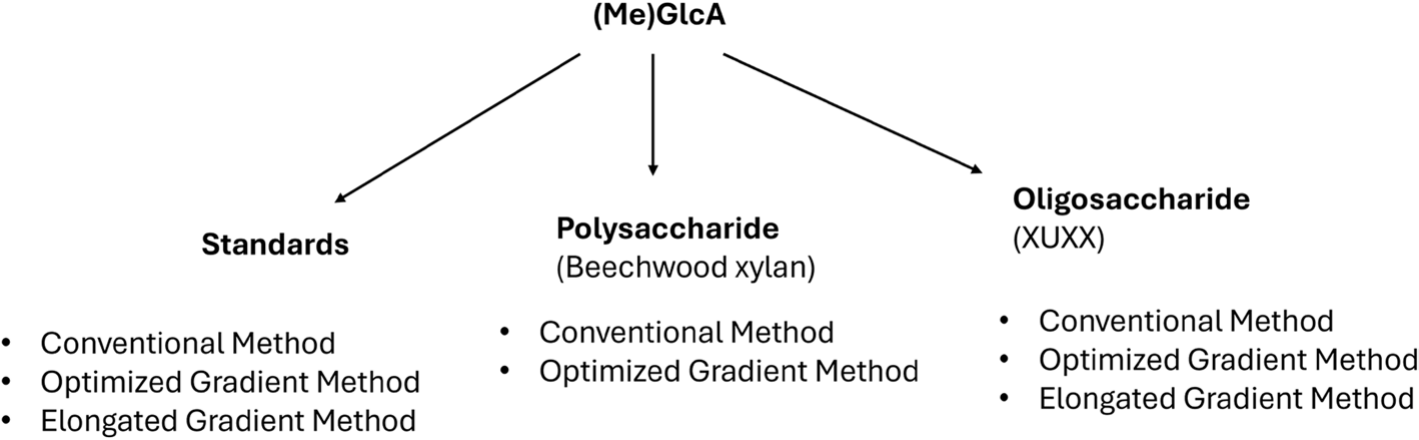
Schematic view illustrating the three types of analytes used in this study and the various methods applied for their analysis

Enzymatic hydrolysis of Beechwood xylan as a polysaccharide substrate and XUXX as an oligosaccharide substrate, was performed using a GH115 α-Glucuronidase (0.01 g/L) [14] The aim was to assess the activity of the enzyme on (Me)GlcA branches presented in both a polysaccharide (beechwood xylan) and an oligosaccharide (XUXX). To validate this concept, it was necessary to confirm release of the (Me)GlcA branch, which was verified by ESI–MS (below). Three analytical methods, including the previously proposed with isocratic elution [24] (termed conventional) and two optimized gradient methods developed here, were performed for the detection and analysis of the different reaction mixtures. Figure 1 illustrates the scheme detailing the respective sample, and the analytical methods employed for each substrate and reference compound (standard).

### Analysis of hardwood-related reference compounds

**Fig. 2:**
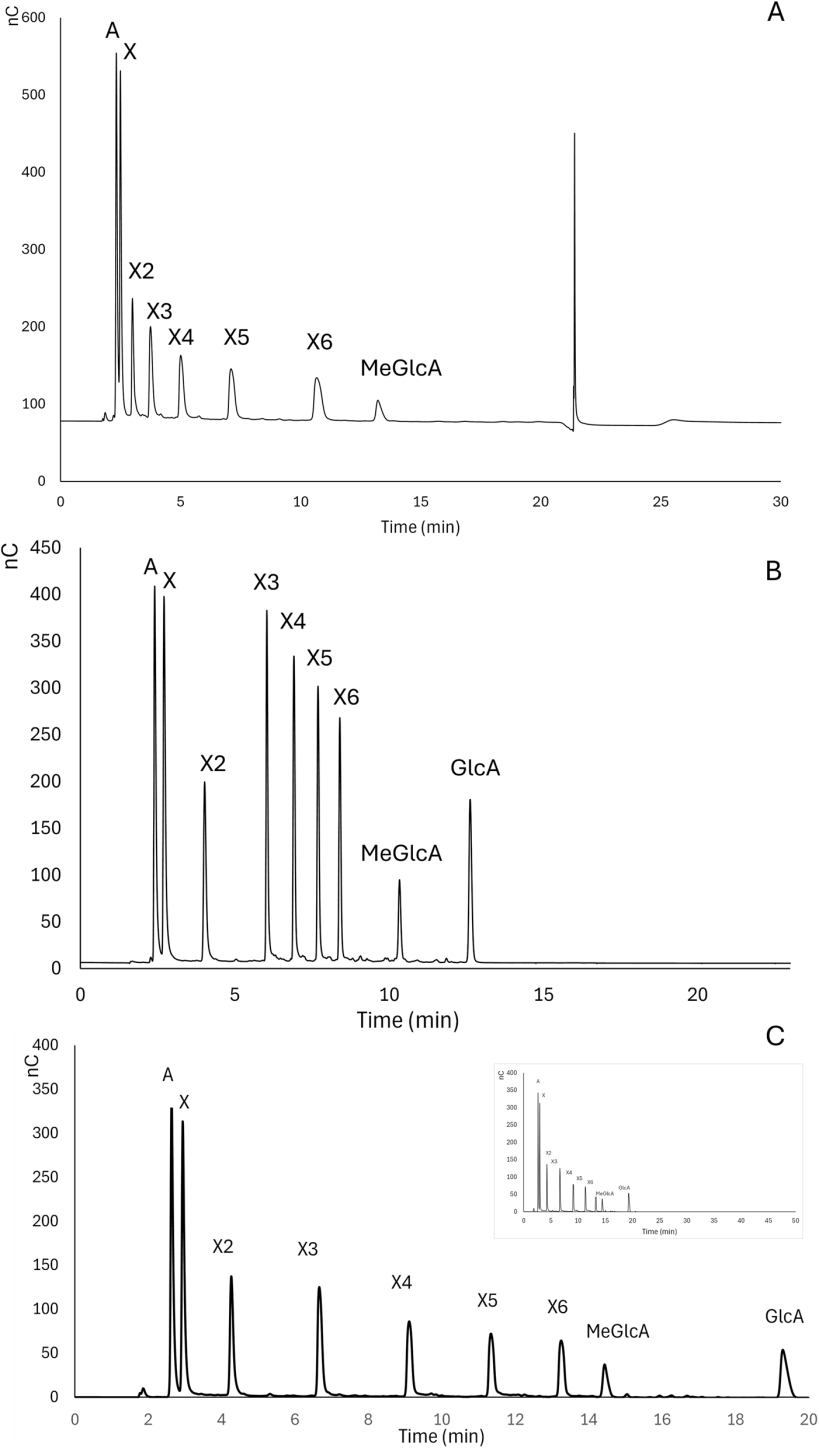
Separation of hardwood-related reference compounds with different analytical methods. **A** presents the conventional method, with total analysis time of 30 min. **B** presents the optimized gradient method with total analysis time of 23 min. **C** presents elongated gradient method with total analysis time of 50 min. To facilitate the comparison among the elution of different compounds a zoomed-in chromatogram of the first 23 min, is shown in C, the overall run is displayed in the upper right corner

To assess the performance of both the conventional and newly optimized methods, analysis of hardwood-related reference compounds consisting of standard mixtures of arabinose (A), xylose (X), xylobiose (X2), xylotriose (X3), xylotetraose (X4), xylopentaose (X5), xylohexaose (X6), methyl-glucuronic acid (MeGlcA), and glucuronic acid (GlcA) was first carried out (Fig. 2) using the approaches detailed in Fig. 1.

As seen in Fig. 2A, arabinose and xylose were not completely separated using the conventional method, although separation of oligoxylosaccharides was successful. MeGlcA eluted at a retention time of 13 min during isocratic conditions, while GlcA elution was delayed to a later time and could not be resolved, as it potentially overlaps with the alkaline peak from the washing step, at a retention time of 21.3 min.

The optimized gradient method was initially performed using a total running time of 23 min. Under these conditions, all compounds (monosaccharides, oligosaccharides, MeGlcA, and GlcA) were detected, although the monosaccharides eluted very close to each other. The MeGlcA and GlcA eluted at retention times of 10.2 min and 12.7 min, respectively, where the concentration of NaOAc was approximately 100 mM (Fig. 2B).

Following this, the total run time was extended to 50 min using the elongated gradient method with a slow gradient increase, resulting in enhanced separation of the compounds of interest (Fig. 2C). MeGlcA eluted at 14.4 min, while GlcA eluted at 19.2 min, with NaOAc concentrations of approximately 60 mM and 80 mM, respectively (Fig. 2C). Based on the successful separation of the reference compounds using both the gradient and elongated gradient methods, their application to galacturonic acid and monosaccharides was tested. Both methods successfully separated MeGlcA, galacturonic acid, and GlcA in that order of elution (Supplementary Fig. 1). However, the methods were less effective for separation of monosaccharides, resulting in co-elution of the different sugars (Supplementary Fig. 2 & 3).

### Analysis of released (Me)GlcA branch from enzymatic hydrolysis of polymeric beechwood xylan

**Fig. 3:**
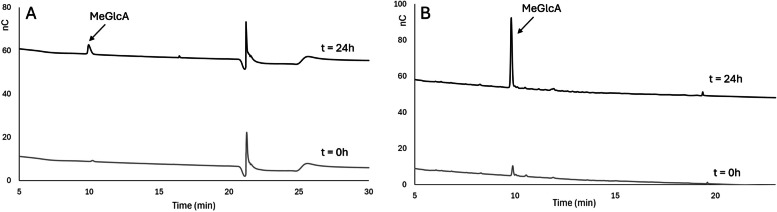
Chromatograms illustrating the detection of the released (Me)GlcA branch from enzymatic hydrolysis the polymeric beechwood xylan at t = 0 and t = 24h using (**A**) the conventional method, and **B** the optimized gradient method

Following the analysis of the standard reference compounds, the (Me)GlcA branch released from enzymatic hydrolysis of polymeric beechwood xylan 5 g/L was analyzed using both the conventional method (Fig. 3A) and the optimized gradient method (Fig. 3B).

As seen in Fig. 3A, use of the conventional method, resulted in only one peak that appeared at the retention time of 10 min. The peak at 21.5 min was attributed to the high concentration of NaOAc used to remove impurities from the column. The same sample was also analysed using the optimized gradient method, which yielded a single product peak at a retention time of 9.2 min (Fig. 3B). In comparison to the conventional method, the peak was more distinct, attributed to the higher NaOAc concentration used in the method. Notably, there was no peak observed at the retention time of 21.5 min. The polymeric Beechwood xylan was not detected due to its high degree of polymerization, which exceeded the capabilities of the column and method employed.

**Fig. 4:**
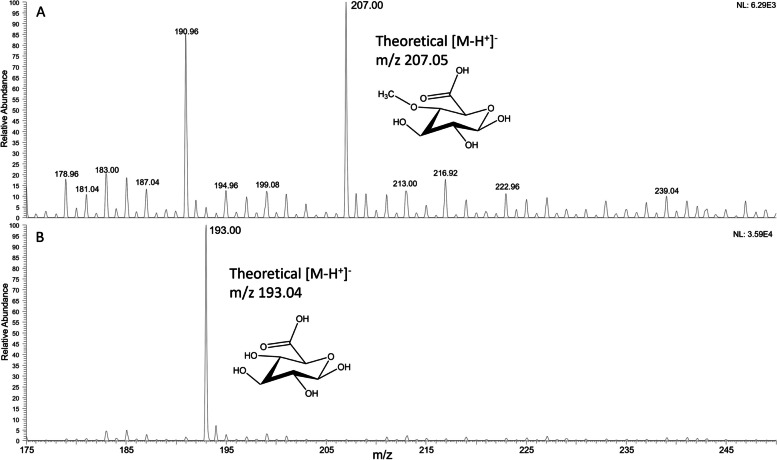
ESI–MS spectra of (**A**) the fraction collected sample from the HPAEC-PAD, and **B** 10 μM of standard GlcA

The detected peak was expected to be MeGlcA and to confirm this, the peak was isolated using the fraction collector feature of the autosampler in the HPAEC-system and analysed by ESI–MS. Figure 4A shows the MS spectra of the collected fraction after background subtraction. The most intense signal corresponded to a monoisotopic ion of m/z 207.0, indicating MeGlcA [M-H^+^]^−^. A GlcA standard was also analysed and compared with the mass spectra of the detected peak (Fig. 4B).

### The consistency of the optimized gradient method for analyzing the kinetics of enzymatic hydrolysis of the beechwood xylan

**Fig. 5:**
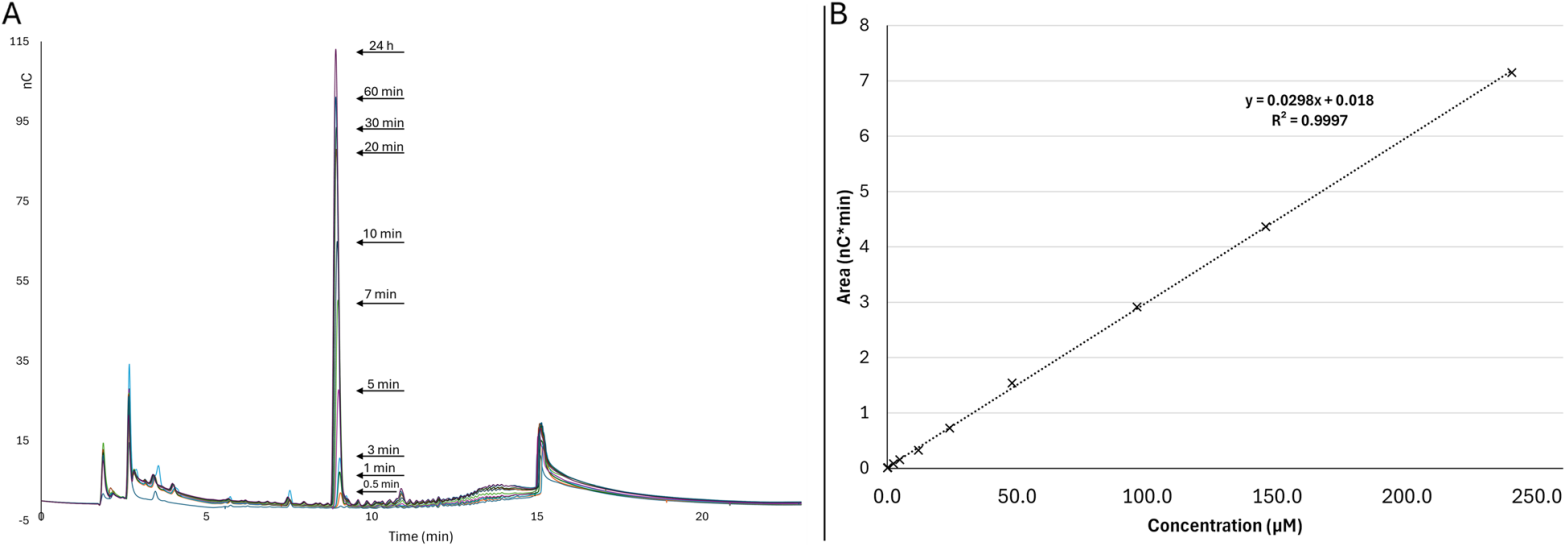
**A** Enhanced MeGlcA peak resulting from enzyme hydrolysis of Beechwood xylan (**B**) Standard curve of MeGlcA plotted using a first-order linear equation (*n* = 2)

To assess the reproducibility of the optimized gradient method and the rate of the reaction, samples were collected at regular intervals and analyzed. Overlay of the chromatograms revealed an increasing MeGlcA peak as the reaction progressed (Fig. 5A). Hence, the method shows promise for quantification and calculation of kinetic parameters. Moreover, a reference compound of MeGlcA was analysed at various concentrations using the same method. A total of 10 concentrations were tested ranging from 0.1—240.2 μM which were used to construct a standard curve (Fig. 5B). The limit of quantification (LOQ) was determined to be 0.1 μM, as it was found to be approximately 9 times higher than the noise level of the base line. Throughout the 10 different MeGlcA concentrations used to construct the standard curve the R^2^ value was 0.9997 suggesting a linear response to the analyte in this concentration range (Fig. 5B).

### Analysis of released (Me)GlcA branch from enzymatic hydrolysis of the oligosaccharide XUXX

**Fig. 6:**
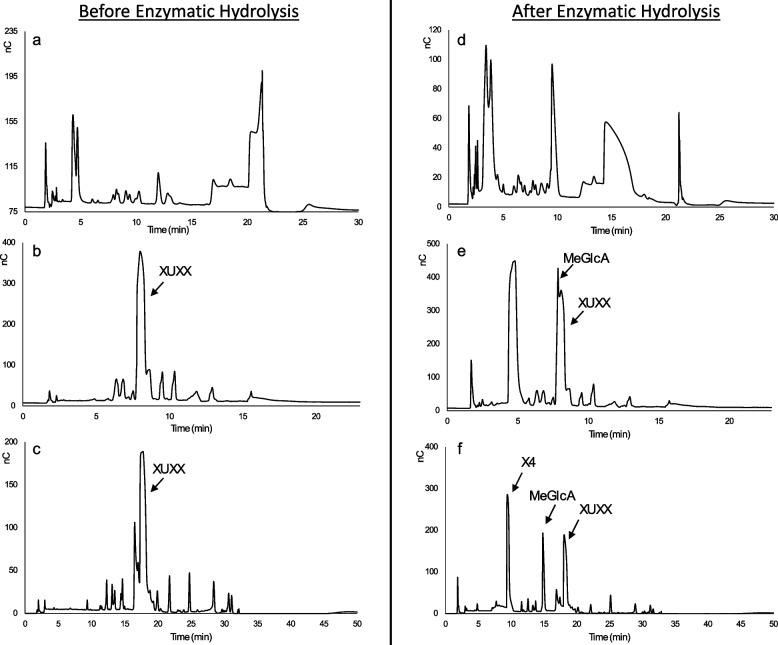
The HPAEC-PAD profiles of the reaction mixture before and after enzyme hydrolysis using (**a**, **d**) the conventional method, **b**, **e** the optimized gradient method, and **c**, **f** the elongated gradient method

The oligosaccharide, XUXX 5 g/L, underwent enzymatic hydrolysis, and samples were collected before (0 h) and after (24 h) incubation with the enzyme, and were subsequently analyzed using the conventional, optimized gradient, and elongated gradient methods (Fig. 1). The HPAEC-PAD profiles of the substrate and products before and after hydrolysis are shown in Fig. 6.

The HPAEC-PAD profile of XUXX before enzymatic hydrolysis showed multiple peaks throughout all three methods (Fig. 6 a-c), indicating a relatively low purity of the substrate. The highest peak was assumed to represent XUXX, a hypothesis supported by subsequent chromatograms of the substrate after enzyme hydrolysis (best visible in Fig. 6 f), where the peak area decreased.

The HPAEC-PAD profiles of the reaction mixture after enzyme hydrolysis indicated inconclusive chromatograms when using the conventional method (Fig. 6a and d). Despite the observable impact of the enzyme on the substrate, evidenced by the reduced solubility of the reaction mixture (ocular), the resulting products could not be detected (Fig. 6 d).

The optimized gradient method improved the separation of the reaction mixture both before and after enzyme hydrolysis. However, the method proved inadequate for detecting and quantifying MeGlcA, as XUXX and MeGlcA eluted at similar retention times with overlapping baselines (Fig. 6b and e).

To resolve this issue, the total running time of the optimized gradient method was extended. Consequently, the gradient (B-C; NaOAc-NaOH) was increased to 50%, at a slower rate, which increased the retention of the compounds (Fig. 6c and f). The extended gradient improved the separation within the reaction mixture, particularly after 10 min, resulting in clear peaks for both X4 and MeGlcA. This improvement allowed better detection and quantification of the products.

Although some compounds still co-eluted at earlier retention times, they no longer interfered severely with the peaks of the products of interest after enzymatic hydrolysis (Fig. 6f). Furthermore, increasing the gradient steepness could potentially further eliminate interferences.

It is important to highlight that in the elongated gradient method, a complete product profile was obtained within approximately 35 min, both before and after enzymatic hydrolysis. Despite a total method duration of 50 min, the primary objective was to achieve a reliable method with stable baseline, starting at 0 nC for subsequent injections. It is likely that this objective could still be accomplished within a shorter timeframe if the washing step was optimized.

## Conclusion

This study developed an advanced HPAEC-PAD method for analyzing the products resulting from enzymatic hydrolysis of polymeric hardwood xylan and the oligosaccharide XUXX, specifically targeting (Me)GlcA, all within a single analytical run. The conventional method using isocratic elution established by Basumallick and Rohner [24] failed to adequately separate all hardwood-related reference compounds and oligosaccharides, and to overcome these limitations, we propose a gradient method using NaOAc, which effectively eluted all hardwood-related reference compounds in a single run. A standard curve of MeGlcA assured first order linearity in a wide range of concentrations, making the method excellent for quantification. Furthermore, adjustment of the gradient rate enhanced the separation of a number of reference compounds and improved the resolution of oligomeric substrates and products.

## Supplementary Information

Supplementary Material 1.

## Data Availability

Data is provided within the manuscript file. All other data and materials supporting the findings of this study are available from the corresponding author upon reasonable request.

## Abbreviations

A: Arabinose
CID: Collision-induced dissociation
ESI-MS: Electrospray Ionization Mass Spectrometry
GlcA: Glucuronic acid
GX: Glucuronoxylan
HPAEC-PAD: High performance anion exchange chromatography with pulsed amperometric detection
LOQ: Limit of quantification
(Me)GlcA: Methylglucuronic acid and/or glucuronic acid
MeGlcA: Methylglucuronic acid
MGX: Methylglucuronoxylan
MS: Mass Spectrometry
X: Xylose
X2: Xylobiose
X3: Xylotriose
X4: Xylotetraose
X5: Xylopentaose
X6: Xylohexaose
XUXX: 4-O-Methyl-α-D-Glucuronyl- xylotetraose
