# Supplementary material for: An improved HPAEC-PAD method for the determination of D-glucuronic acid and 4-O-methyl-D-glucuronic acid from polymeric and oligomeric xylan
Source: BMC Biotechnol. 2024 Dec 12;24:100. doi: 10.1186/s12896-024-00931-9 (PMC11636049; doi:10.1186/s12896-024-00931-9)
Supplement: Supplementary file 1 — Supplementary Material 1. [file 12896_2024_931_MOESM1_ESM.docx]

# **An improved HPAEC-PAD method for the determination of D-glucuronic acid and 4-O-methyl-D-glucuronic acid from polymeric and oligomeric xylan**

Savvina Leontakianakou*, Carl Grey, Eva Nordberg Karlsson, Roya R.R. Sardari

Division of Biotechnology, Department of Chemistry, Lund University, PO Box 124, Lund SE-22100, Sweden

* Corresponding author: S. Leontakianakou: [savvina.leontakianakou@biotek.lu.se](mailto:savvina.leontakianakou@biotek.lu.se)

Figure 1. Separation between MeGlcA, Galacturonic acid and GlcA demonstrating successful separation of all three uronic acids with the gradient method.

Figure 2. Overlayed chromatograms of individually analysed monosaccharides show that all compounds are detected, but neutral monosaccharides are poorly separated. Suggesting the method is more suitable for charged molecules and oligosaccharides.

Figure 3. HPAEC-PAD profile of a mixture of reference compounds (rhamnose, arabinose, galactose, mannose, glucose, and xylose) shows that only rhamnose and arabinose are separated, though their baselines overlap. All other compounds elute at approximately 2.75 min.
